# Prediction of lithium-ion battery SOC based on the fusion of MHA and ConvolGRU

**DOI:** 10.1038/s41598-023-43858-5

**Published:** 2023-10-02

**Authors:** Pei Tang, Jusen Hua, Pengchen Wang, Zhonghui QU, Minnan Jiang

**Affiliations:** School of Automotive Engineering, Yancheng Institute of Technology, Yanchen, 224051 China

**Keywords:** Fuel cells, Batteries

## Abstract

If the charging state of the lithium-ion battery can be accurately predicted, overcharge and overdischarge of the battery can be avoided, and the service life of the battery can be improved. In order to improve the prediction accuracy of SOC, a prediction method combined with convolutional layer, multi-head attention mechanism and gated cycle unit is proposed to extract data feature information from different dimensions of space and time. Using the data set of the University of Maryland, we simulated the battery in real vehicle operating conditions at different temperatures (0 °C, 25 °C, 45 °C). The test results showed that the mean absolute error, root mean square error and maximum prediction error of the model were 0.53%, 0.67% and 0.4% respectively. The results show that the model can predict SOC accurately. At the same time, the comparison with other prediction models shows that the prediction accuracy of this model is the highest.

## Introduction

Lithium-ion batteries have become the preferred battery type for electric vehicles due to their large capacity, environmental friendliness and higher energy, but safety concerns also arise with their use^1^. Inaccurate estimates of the battery's state of charge (SOC) prevent vehicle owners from accurately predicting remaining mileage. Inaccurate SOC values can also cause overcharging and discharging, which can damage internal chemical materials, leading to reductions in the battery's lifetime^2^. Since current measurement devices cannot directly measure SOC, accurate prediction of battery charge state is necessary. Currently, SOC prediction methods are mainly divided into three categories: 1. traditional estimation methods; 2. model-based estimation methods; 3. data-based forecasting methods^3^.

Traditional estimation methods include ampere-hour integration method and open-circuit voltage method^4^. Ampere-hour integration method^5^ suffers from large errors due to measurement errors, especially due to current offset errors, and introduces errors during initialization that cannot be compensated during subsequent processes. Battery capacity also depends on temperature and age status, which is difficult to correct. Although open-circuit voltage measurements have high estimation accuracy, they require long periods of inactivity (hours, even days) to achieve thermodynamic equilibrium of the battery, making them unsuitable for real-time measurement of vehicle SOC. The method of combining the ampere-hour integration method and the open-circuit voltage method is proposed by Yuan xu^6^ to estimate the battery SOC value. The correspondence between the open circuit voltage and the battery SOC is determined through the relevant charge and discharge experiments, and the relationship between the time required for the battery to achieve full standing and the battery SOC, so as to accurately estimate the initial value of the battery SOC, and then quickly estimate the battery SOC value in real time based on the ampere-hour integration method.

The model-based estimation method requires the establishment of complex equivalent circuit models for lithium-ion batteries and the use of algorithms such as Kalman filter to estimate SOC online^7^. The model-based SOC estimation method can calibrate the estimated value in real time, thereby the SOC real-time estimation accuracy is greatly improved. However, as the battery ages or the temperature changes greatly, the model parameters of the battery will be changed at the same time so that the accuracy of the model and the estimation accuracy of SOC is reduced. Shijie Li^8^chose the unscented Kalman filter (UKF) algorithm to estimate the SOC, it is found that with the increased of the initial value deviation of SOC, the error of SOC estimation increases in the initial period, but after the error converges, the estimated error of SOC can still be maintained within 2%. SOC estimation of temperature and discharge rate compensation introduced by Jiao Zhen^9^. The common SOC estimation algorithms are analyzed and compared, and the method of using the EKF algorithm combined with the second-order RC circuit as the power estimation method is established, and the steady-state, dynamic and initial value automatic correction simulation verification is established. In addition, the temperature compensation factor and discharge rate compensation factor are added to the EKF algorithm, and the SOC estimation accuracy of the battery is improved by the comparison of simulation verification.

Data-based prediction methods only require learning the relationship between SOC of lithium-ion batteries and discharge data, avoiding the difficulty of determining the initial value in other prediction methods and eliminating the accumulation error problem with higher prediction accuracy^10^. With the widespread application of convolutional neural networks (CNN) and recurrent neural networks (RNN) in many fields, data-based prediction methods have also been used in the prediction of battery SOC. RNN can effectively preserve historical input information and has temporal memory capability, but the Simple Recurrent Neural Network (SimpleRNN) loses information with increasing time steps. Long short-term memory (LSTM) neural network relies on the input of past samples and effectively solves the problem of SimpleRNN unable to capture long-term dependencies. Shuiping Ni^11^ proposed the CNN-LSTM method for predicting battery SOC by combining CNN and LSTM, and the experimental results showed that the model has accurate and stable battery SOC prediction effects. Yanwei Wu^12^ used the gated recurrent unit (GRU) to establish an SOC prediction model and simulations demonstrated that the GRU prediction model had a promising performance for estimating SOC. Chong Wen^13^ proposed a data-based predictive method for lithium-ion battery SOC based on the enhanced recurrent neural network algorithm with attention mechanism, which can improve the accuracy of SOC estimation results by reducing prediction errors.

In this paper, we propose the ConvolGRU-MHA method for predicting the SOC of lithium-ion batteries, which combines Convolutional layer, GRU and multi-head attention mechanism (MHA). The multi-head attention mechanism (MHA) overcomes the shortcomings of single-head attention in processing complex models and can better extract feature information from multiple aspects to prevent model overfitting. The convolutional layer (Convol) is the most important part of a convolutional neural network because it can extract data features. GRU can save the time sequence features of important data well, making it a good candidate for SOC prediction. We use MHA to improve the feature selection process and adopt ConvolGRU to calculate representative features from the battery voltage data. Finally, we use the estimated result of the sequence data to predict the SOC of the lithium-ion battery.

## ConvlGRU combined with MHA for state of charge prediction model

### General model structure

**Figure 1:**
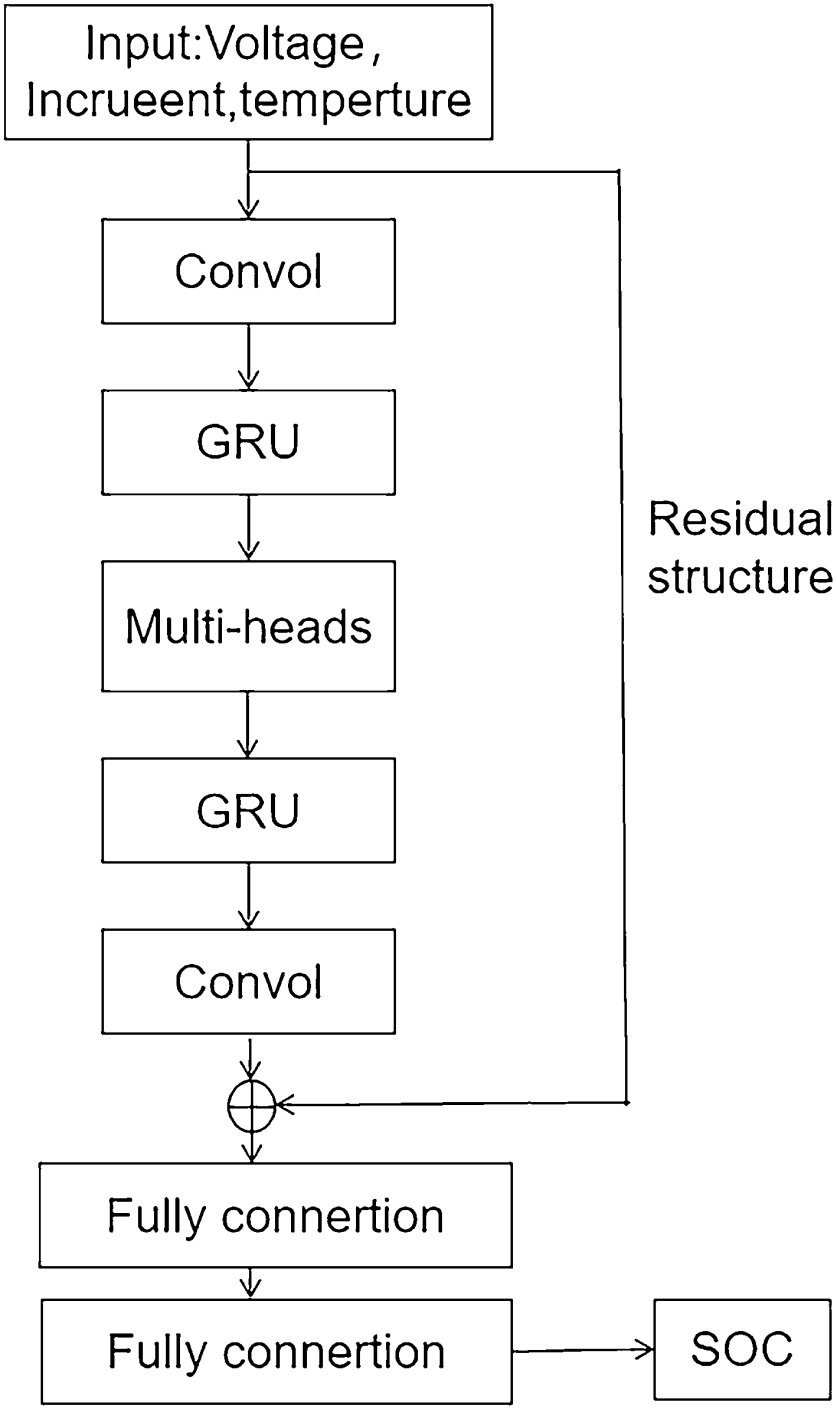
Structure diagram of the predictive model.

In this study, we propose a model that combines the advantages of Convolutional layers, Gated Recurrent Unit (GRU) networks, and Multi-Head Attention Mechanism (MHA) to create the ConvolGRU-MHA fusion model, as shown in Fig. 1. First, the input channel is amplified by the convolution layer to extract features from the input data. Next, the GRU network extracts time-series information features and discards unimportant feature data to improve network performance. Multi-head attention mechanism is then used to extract data features from various levels to prevent the model from overfitting. GRU network and convolutional layer are added to ensure the output's dimension is consistent with the input's dimension, enabling the addition of residual structures to guarantee the network's performance. Finally, two fully connected layers output the predicted SOC value.

### Convolutional neural networks

**Figure 2:**
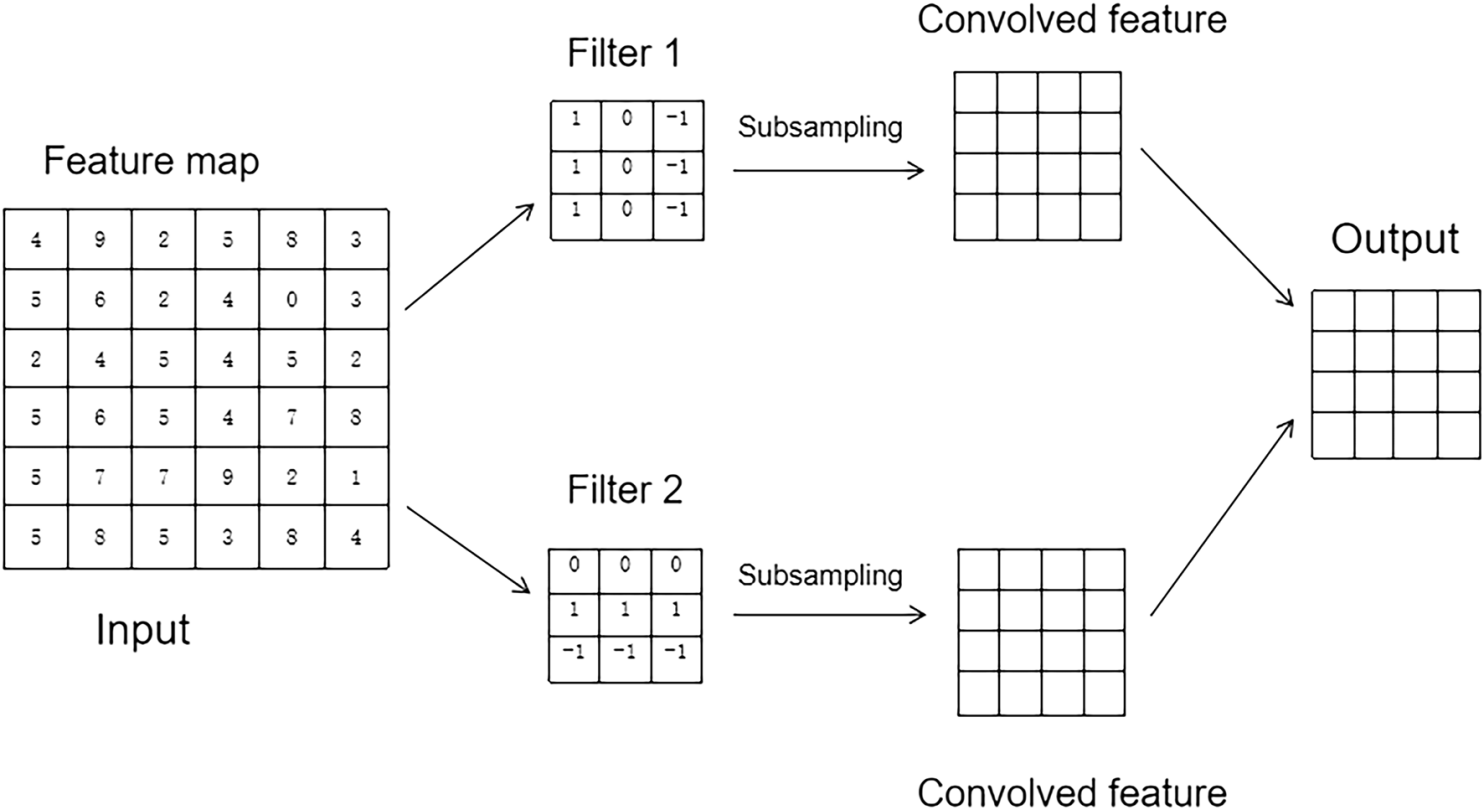
A simple convolutional neural network example model^15^.

Convolutional layers are not only capable of extracting features from inputs but also of extracting information from time series data, making them particularly suitable for extracting data with temporal features such as those found in batteries^14^. A simple convolutional neural network model is shown in Fig. 2. The convolution of 1 × 1 is chosen in this paper, whose direct effect is to broaden the number of channels, so that the prediction model can continuously extract the features of the data, and each layer can learn more abundant features, and the prediction effect will be improved accordingly.

### Structure of GRU

**Figure 3:**
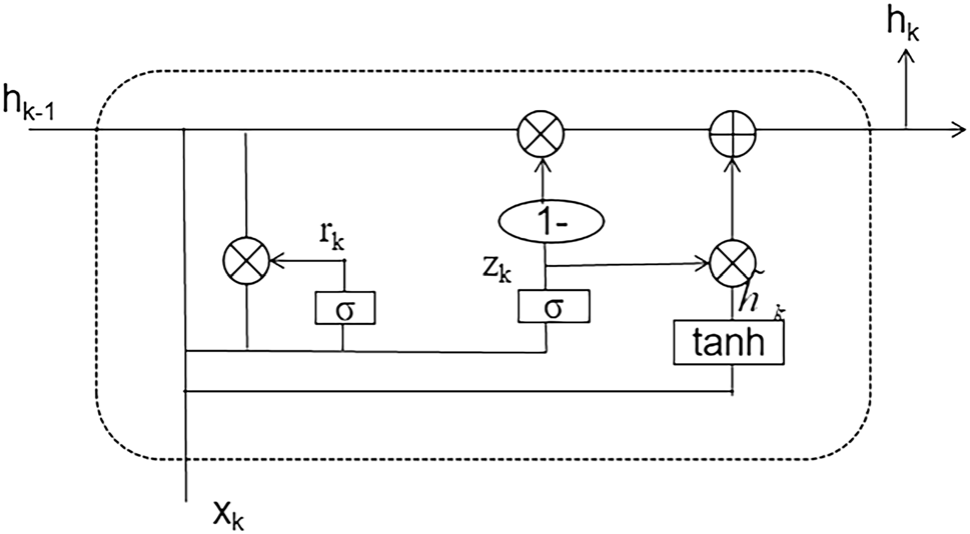
GRU structure diagram.

In contrast to the Long Short-Term Memory (LSTM) structure, which contains many parameters, Gated Recurrent Units (GRU) Structure is simple and contains fewer parameters while still using two gate structures: reset and update gates^16^. These gates combine input parameters, previous states, and hidden states to control the output information. Consequently, the GRU structure can train the model faster. Figure 3 shows the GRU structure diagram.

The computational formula is shown in Eqs. (1, 2, 3, 4):

1 $$z(k) = s\{ W_{Z} \times [h(k - 1),x(k)]\}$$

2 $$r(k) = \sigma \{ W_{r} \cdot [h(k - 1),x(k)]\}$$

3 $$\tilde{H}({\text{k}}) = \tanh \{ W_{{\tilde{H}}} \cdot [r(k)*H(k - 1),x(k)]\}$$

4 $$H(k) = [1 - z(k)] \times H(k - 1) + z(k) \times \tilde{H}({\text{k}})$$

z(k) and r(k) are the states of the reset and update gates, respectively; h(k) is the output;Wz, Wr, and Wh represent the reset gate, update gate, and output weight, respectively.The reset gate checks whether past data has value for predicting future information. If not, its value approaches 0, and the model forgets the past information and saves the current input information^17^. If the model considers past information to be related to future information, the reset gate value approaches 1, and it is added to the current information.

### Multi-heads attention mechanism

**Figure 4:**
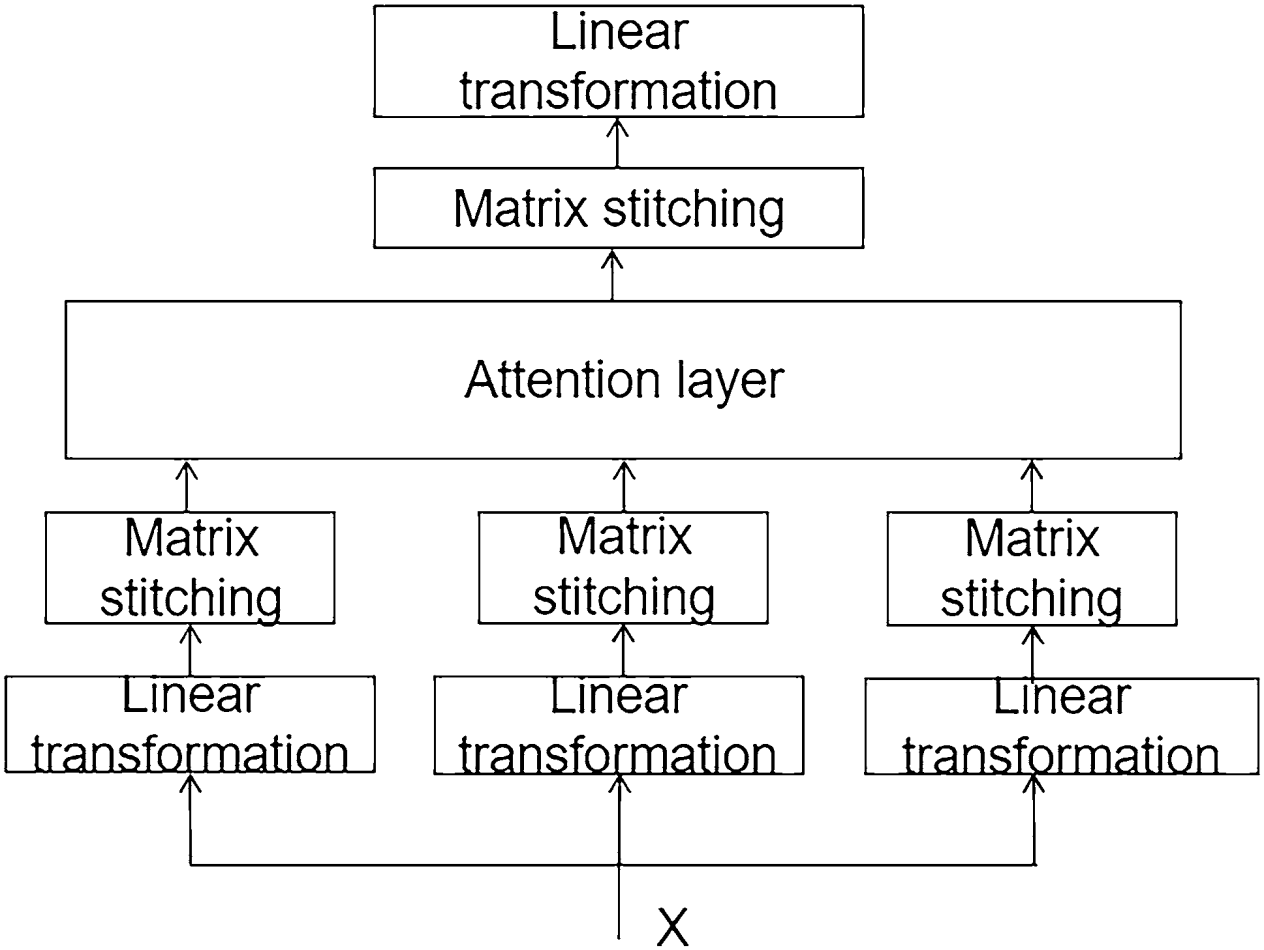
Model of multi-head attention mechanism.

Multi-heads Attention Mechanism can be useful to allocate more computational resources to the information that is most important for the current task, whereas other information is allocated fewer resources to increase the computational efficiency and accuracy of the model^18^. In GRU, features that depend on each other at long distances require accumulation of information over multiple time steps which may result in diminished effective feature capture as the distance increases. Introduction of Multihead Attention Mechanism also can more easily capture long-distance dependent features in the sequence. In the calculation process, any two features in the sequence can be directly linked together via a calculating step. Therefore, distant dependent features are directly connected, thereby greatly reducing the distance between them, and facilitating effective utilization of these features and accelerating the convergence of the model. The Multihed Attention Mechanism model is shown in Fig. 4.

### Residual structure

Residual Structure Due to the excessive model, overfitting may occur during the training process, ultimately leading to an increase in the error rate of estimating SOC. To prevent overfitting and ensure the network's stability, additional connection structures, i.e., residual structures, were introduced in the prediction model. The use of residual connections not only enhances the convergence speed of the network but also makes it more stable. Moreover, residual connections do not increase the number of parameters nor increase computational complexity, yet enables the network to learn more valid information, resulting in further reduction of the error rate of the prediction model. Since residual connections are used in the network, the input and output dimensions of the network should be the same. The formula for mapping function H (x) is calculated as shown in Eq. (5):

5 $$H(x) = x + F(x)$$

In Formula (5), x is the input feature of the current residual module; F(x) is layer convolution, activation and other operations^19^. The residual connection does not add extra parameters or computational complexity, but it can help the network model learn more effective information, so as to further reduce the error rate predicted by the model.

Multi-heads Attention Mechanism and residual structure algorithms offer several advantages. Firstly, they can adaptively select data features relevant to SOC to train the network model. The use of Multi-heads Attention Mechanism increases the diversity of feature extractions, and cooperation among multiple heads helps the network learn deeper-level data features. Secondly, residual connections with weight matrices can make the network more stable and robust, and when combined with convolutional neural networks, enhance the accuracy of estimating SOC, and finally, the multi-head parallel processing can accelerate the network's training speed, making the network more responsive to real-time requirements.

## Experimental data and evaluation metrics

### Data source

The data used in this study was obtained from the Center for Advanced Life Cycle Engineering (CALCE) at the University of Maryland^20^. The data measured by CALCE was chosen as our training dataset because the INR-18650R data is the authoritative public data set in the field of lithium-ion battery research, measured by rigorous test methods and sophisticated measuring equipment. It is widely used in the research of lithium-ion battery state estimation methods, which provides the possibility to compare the performance of various state estimation algorithms. The research object was the INR18650-20r battery. They studied the FUDS condition and DST condition test under the conditions of 0 °C, 25 °C and 45 °C when the battery was in the initial value of 80% and 50% of the initial value respectively.There are a total of 12 such data sets.

### Data preprocessing

#### Standardized processing

Although predictive models can be trained on unprocessed data, the difference in magnitude of current, voltage and temperature values could make the learning process difficult. To improve the training speed and sensitivity of the algorithm, it is necessary to standardize the input data (current, voltage, and temperature) of the unprocessed training, validation, and test sets such that the state of charge (SOC) of the battery remains in the range of 0–1. The method used for data standardization is shown in equation:

6 $${\text{x}}{^\prime} = \left( {{\text{x}} - \mu } \right)/\sigma$$

where $\mu$ presents the mean and $\sigma$ represents the standard deviation.

#### Window slide

**Figure 5:**
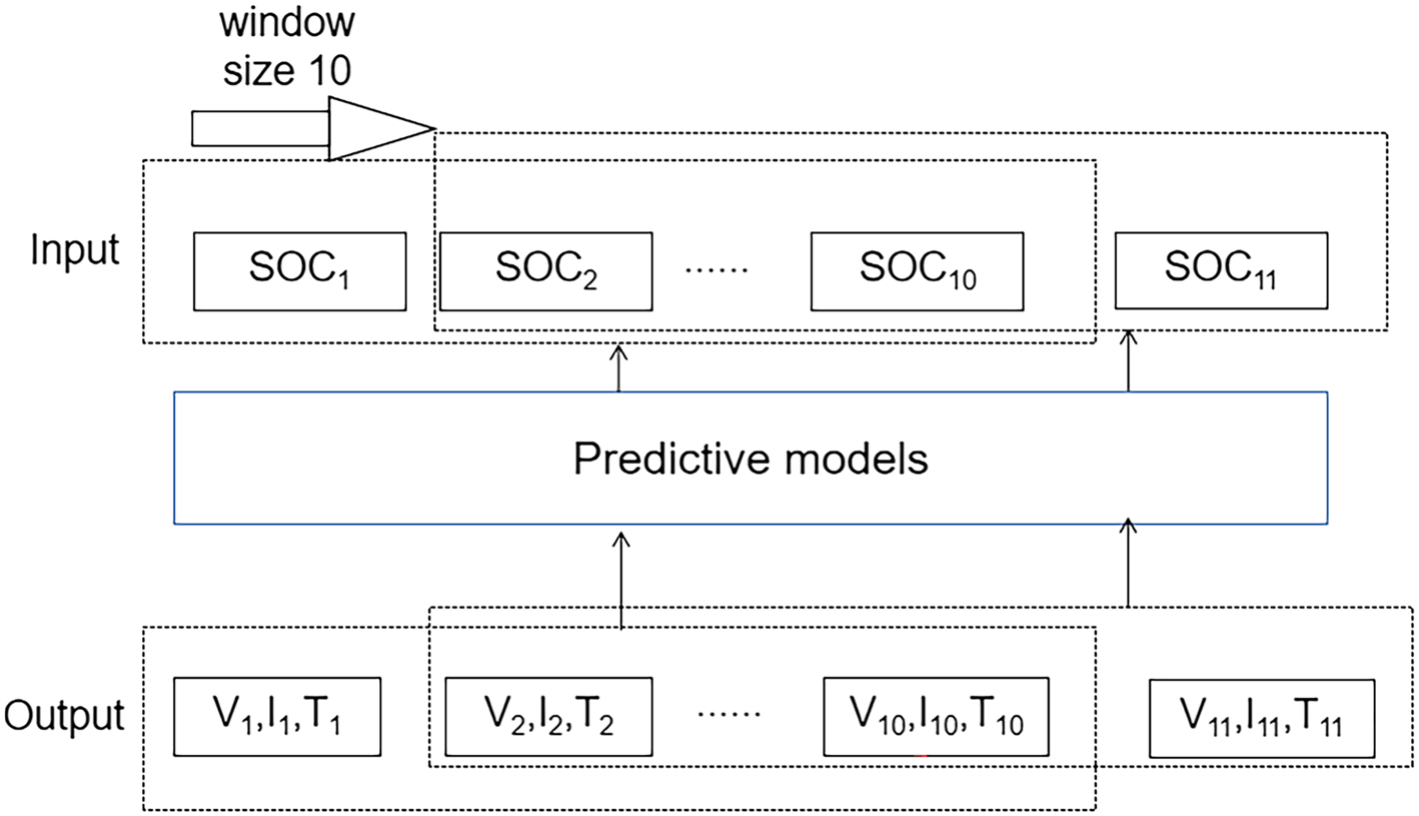
Window slide model.

Window sliding technique is used at the input to include both current and past information in the model, which improves the performance of the predictive model and fully utilizes the temporal information of the battery^21^. The window size is set to 10, which predicts 10-time steps at once with a stride of 1. As the window moves one step forward, 9 pieces of overlapping information are used as input to the model. In the phase of battery decline, more and more relevant SOC characteristics are recorded over time, including voltage, current, temperature and other data. This increases the amount of data available to the model and fully considers the temporal nature of the battery data, which reduces the prediction error of the model. The window sliding structure is shown in Fig. 5.

### Evaluation metrics

To evaluate the performance of the model, two evaluation metrics are selected to assess the predictive performance of the proposed algorithm, including Root Mean Square Error (RMSE) and Mean Absolute Error (MAE). RMSE (Root Mean Square Error) represents the standard deviation of the residual, which is the difference between the predicted and the observed values. RMSE is chosen to indicate the dispersion of the sample. For non-linear fitting, the lower the RMSE, the better. MAE (Mean Absolute Error) represents the average absolute error between the predicted and the observed values. The definitions of RMSE and MAE are shown in Eqs. (7, 8).

7 $$RMSE = \sqrt {\frac{1}{n}\sum\limits_{k = 1}^{n} {(y_{k} - \mathop {y_{k} }\limits^{ \wedge } )^{2} } }$$

8 $$MAE = \frac{1}{n}\sum\limits_{k = 1}^{n} {\left| {\mathop {y_{k} }\limits^{ \wedge } - y_{k} } \right|}$$

where $\mathop {y_{k} }\limits^{ \wedge }$ repsents Predicted value, $y_{k}$ repsents Predicted value, n presents Total number of records.

### Experimental platform and model parameter settings

#### Experimental platform

The experimental environment in this article is Windows 10 64-bit operating system, based on Python 3.8.4 environment programming, all model construction and training are based on pytorch version 1.13. The hardware is configured as an 11th Gen Intel(R) Core(TM) i7-11800H with 32 GB of memory and a GeForce RTX3060 Laptop GPU.

#### Parameter optimization experiments

**Figure 6:**
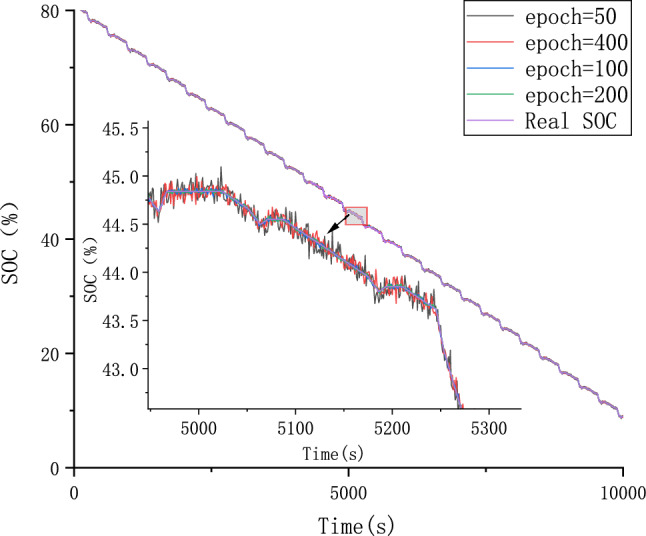
Comparison of different epoch prediction curves.

**Figure 7:**
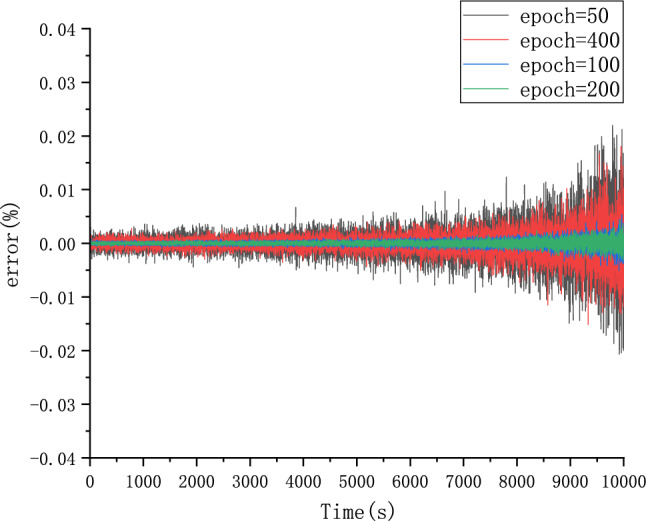
Comparison of different epoch errors.

**Figure 8:**
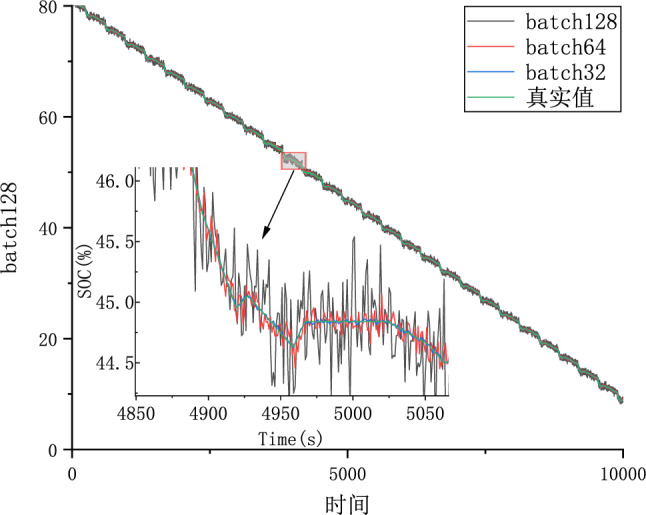
Comparison of different batch size prediction curves.

**Figure 9:**
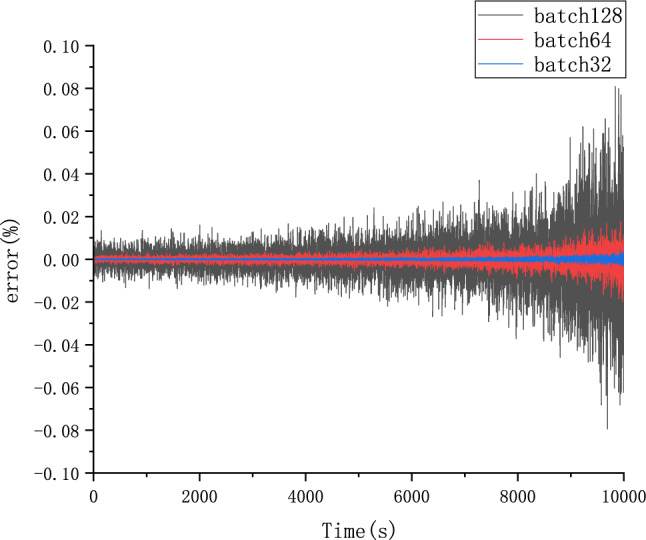
Comparison of different batch size errors.

**Figure 10:**
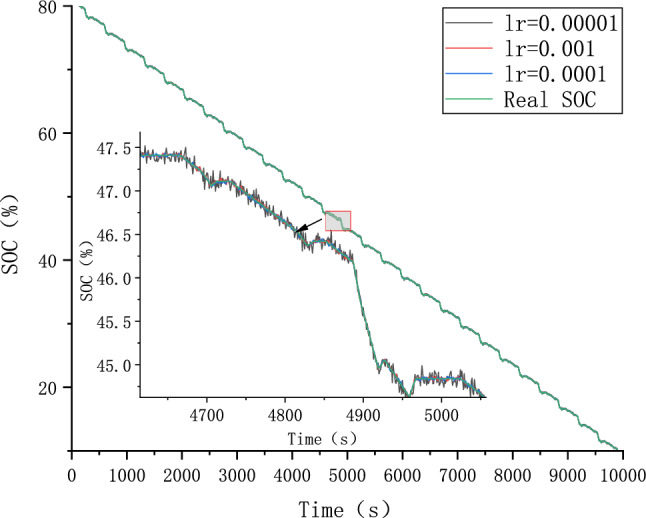
Comparison of different Learning rate prediction curves.

**Figure 11:**
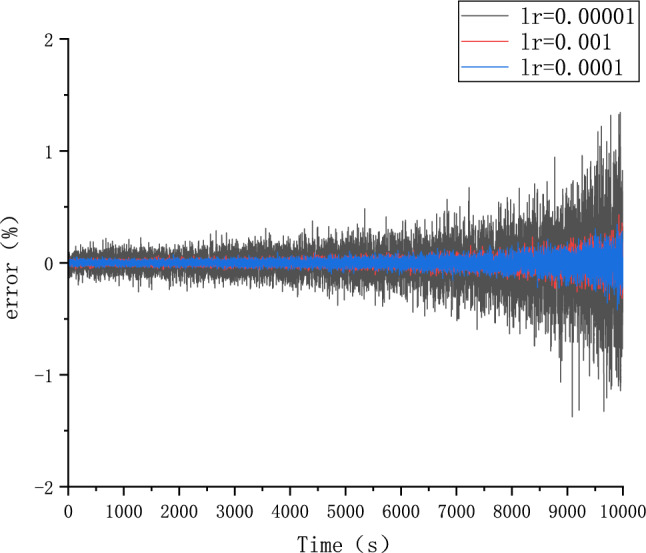
Comparison of different Learning rate errors.

**Figure 12:**
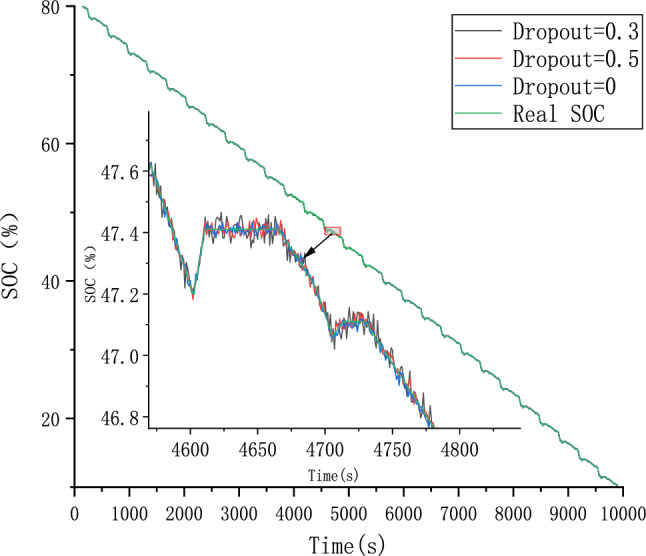
Comparison of different Dropout prediction curves.

**Figure 13:**
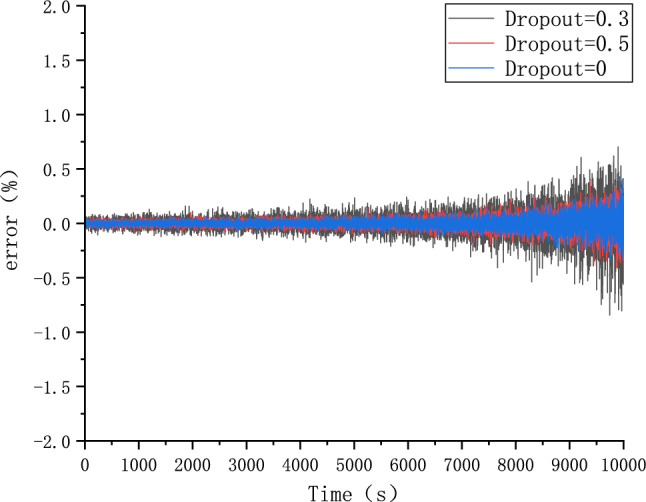
Comparison of different Dropout errors.

Because different parameter Settings will directly affect the prediction effect of the model, it is necessary to carry out parameter optimization experiments to ensure that the model can achieve the best prediction effect. In order to find the optimal model parameters, we conducted comparative experiments on Epoch, Batch_size, Learning rate, Dropout parameters. We use relative error, MAE and RMSE to describe the model prediction effect. The relative error formula is shown in Eq. (9) The prediction effect and relative error of the model are shown in Figs. 6, 7, 8, 9, 10, 11, 12 and 13. Because the curve between the forecast and the real value is too close, we use a local amplification schematic diagram to represent the gap between the predictive value and the real value curve of a certain period of time. The direction of the black arrow refers to a local amplification diagram between the predictive values and the real values in the rectangular range. Finally we selected the parameters with the best effect.

9 $${\text{error}} = \frac{predicted - real}{{real}}$$

Table 1 shows the evaluation index of the prediction effect of the model under each parameter. Through the parameter comparison optimization experiment, we select the parameter with the smallest MAE and RMSE of the model prediction. The parameter settings for the forecast model are shown in the following Table 2:

**Table 1:** Predicted effected of different parameters.

Parameter	Parameter selection	MAE	RMSE
Epoch	50	0.0651	0.0817
	100	0.0123	0.0154
	200	0.0053	0.0067
	400	0.0427	0.0538
Batch_size	32	0.0053	0.0067
	64	0.0639	0.2529
	128	0.2130	0.2901
Learning Rate	0.001	0.0102	0.0126
	0.0001	0.0053	0.0067
	0.00001	0.0418	0.0525
Dropout	0	0.0053	0.0067
	0.3	0.0229	0.0287
	0.5	0.0120	0.0149

**Table 2:** Setting of different parameters in the forecast model.

Parameter	Settings
Loss function	MSE
Epoch	200
Batch_size	32
Learning rate	0.0001
Dropout	0
Optimizer	Adam

## Results and analysis

**Figure 14:**
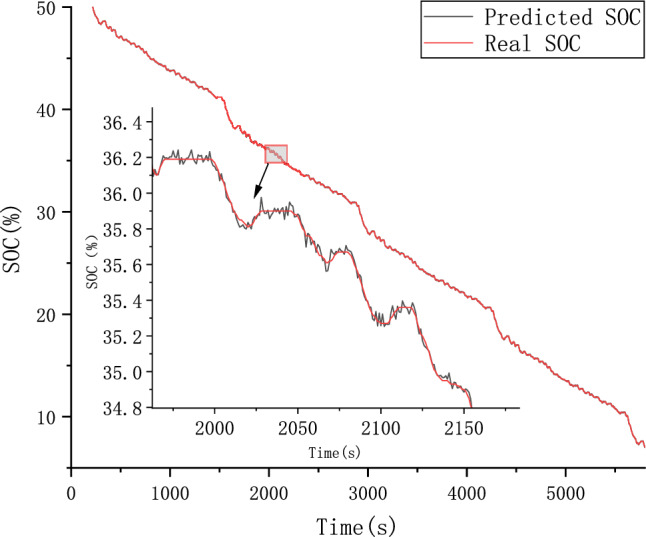
Prediction results at 25 °C and initial 50% SOC.

**Figure 15:**
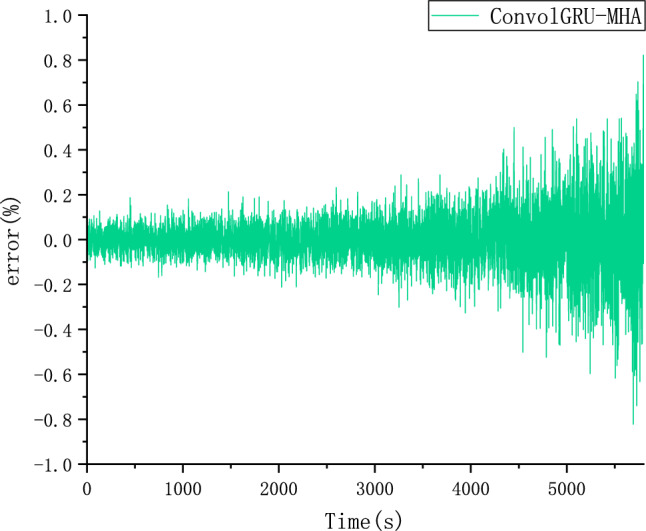
Prediction error at 25 °C and initial 50% SOC.

**Figure 16:**
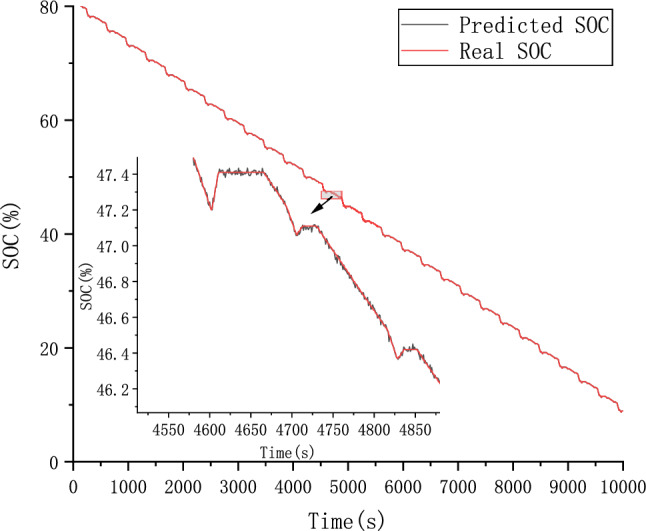
Prediction results at 25 °C and initial 80% SOC.

**Figure 17:**
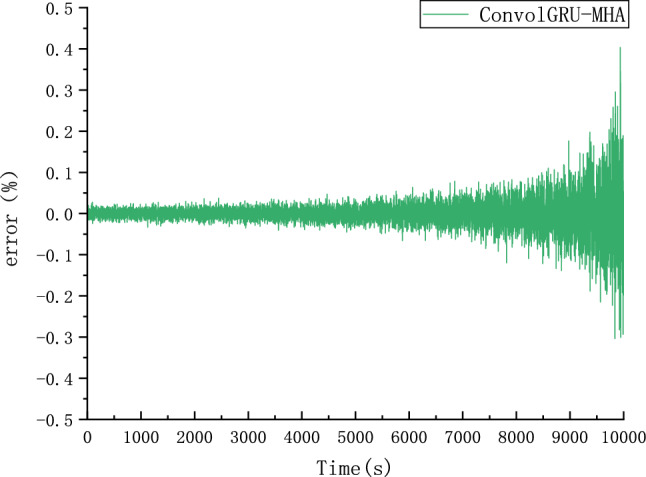
Prediction error at 25 °C and initial 80% SOC.

**Figure 18:**
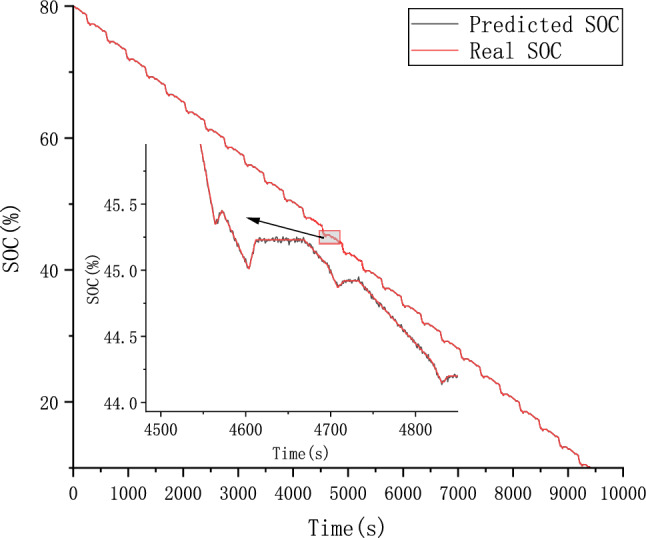
Prediction results at 0 °C.

**Figure 19:**
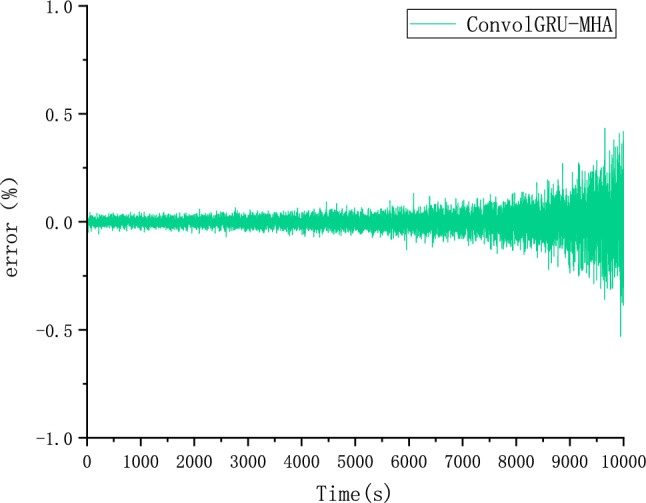
Prediction error at 0 °C.

**Figure 20:**
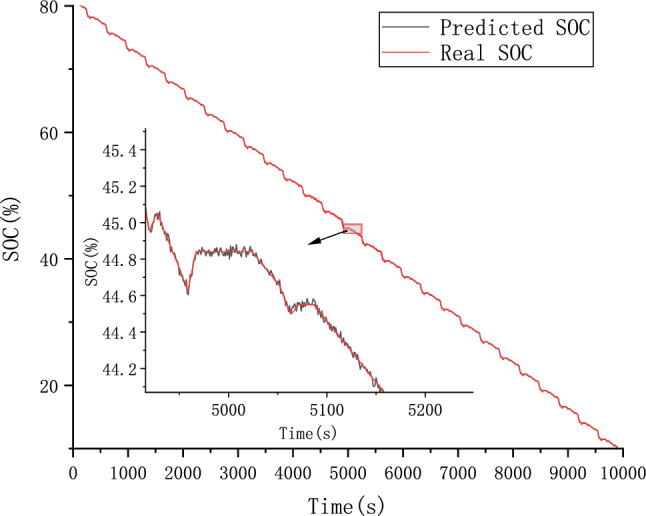
Prediction results at 45 °C.

**Figure 21:**
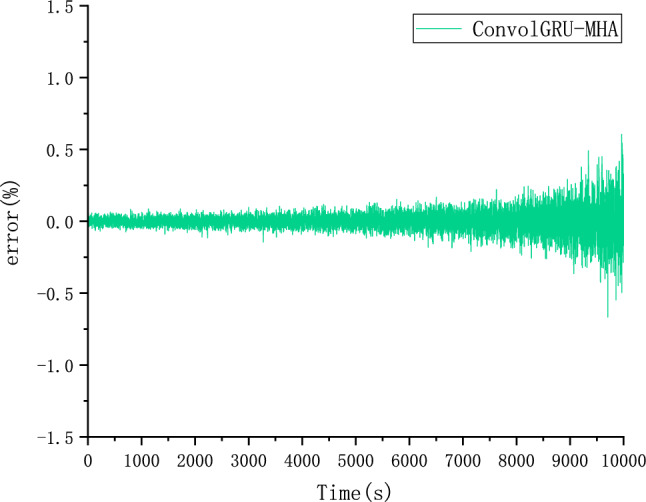
Prediction error at 45 °C.

The CALCE dataset was used as the simulation data in this study. Voltage, current, and temperature were selected as input features of the model and SOC as the output. The performance of the proposed ConvolGRU-MHA SOC prediction model was evaluated using the evaluation indicators RMSE and MAE.In order to explore the effect of initial values on model predictions, we also used the same FUDS operating conditions to predict the battery SOC with initial SOC values of 50% and 80% at 25 degrees Celsius. Figures 14, 15, 16 and 17 compare the prediction curve and the true SOC curve at 80% and 50% of the initial SOC and the prediction error, respectively. The black section represents the predicted SOC values of the model proposed in this study and the red section representing the actual SOC value. AS is shown in Figs. 16, 17, 18, 19, 20 and 21, in order to explore the effect of temperature on model predictions, we select the data recorded by the FUDS conditions of the battery at 0 °C, 25 °C and 45 °C to verify the influence of temperature on the prediction effect of the model.

**Figure 22:**
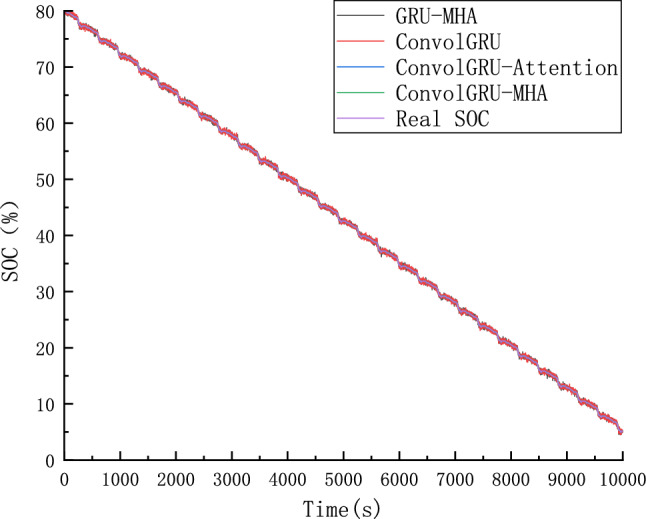
different models SOC prediction results.

**Figure 23:**
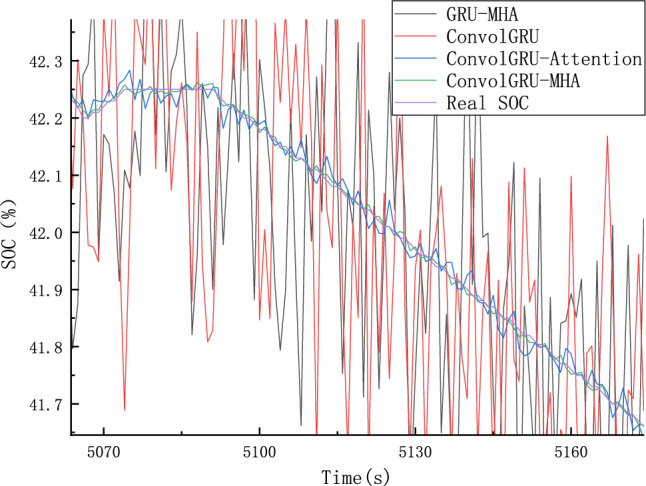
Schematic diagram of local enlargement.

**Figure 24:**
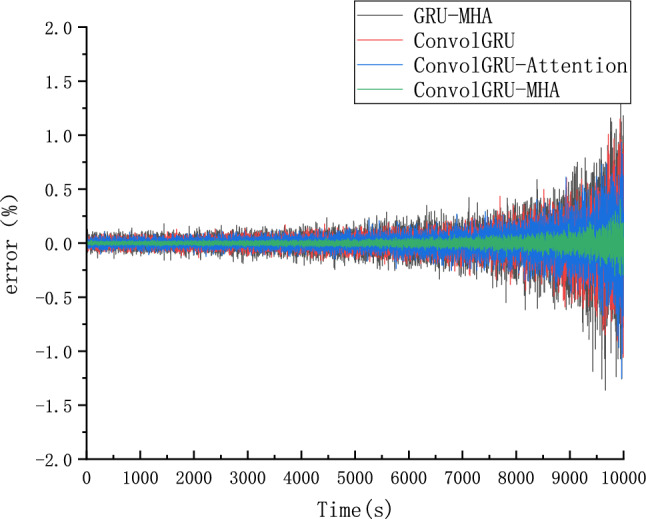
Prediction error of the comparison model.

Four groups of prediction models were employed to predict SOC using ConvolGRU, GRU-MHA, ConvolGRU-Attention, and ConvolGRU-MHA. Predicted results are shown in Fig. 22, and Fig. 23 is a schematic diagram of local magnification. The error between the predicted SOC values and actual values is shown in Fig. 24.As can be seen from the local magnification diagram in Fig. 23, the prediction effect of using convolution layer alone or combining MHA and GRU is inferior to that of using convolution layer, GRU and MHA together. This is because the single combination of GRU and GRU cannot fully extract the features of the data, while the combined method used by the three can extract the feature data from different dimensions of the data, and can use historical data information to improve the prediction accuracy.

From Figs. 14, 15, 16, 17, 18, 19, 20 and 21, it can be seen that regardless of different temperatures or different initial SOC, the model proposed in this paper has good tracking performance and prediction effect, and the maximum error is kept within 1%.

AS is shown from Tables 3 and 4, under different initial values and different temperatures, the evaluation functions RMSE and MAE can prove that the model can adapt to different temperatures and initial SOC values and achieve satisfactory prediction effects.

**Table 3:** RMSE and MAE predicted by SOC of different initial SOC.

Initial SOC (%)	RMSE	MAE
50	0.0138	0.0110
80	0.0067	0.0053

**Table 4:** RMSE and MAE predicted by SOC of different temperature.

Temperature SOC ( °C)	RMSE	MAE
0	0.0111	0.0089
25	0.0067	0.0053
45	0.0138	0.0102

As shown in Figs. 22 and 23, compared with the ConvolGRU, GRU-MHA, ConvolGRU-Attention, and other prediction models, the GRU-MHA and ConvolGRU models exhibit larger fluctuations in both predicted and actual SOC values, while the ConvolGRU-Attention model fused with Convolutional and attention mechanisms has a smoother and more stable predicted curve. The proposed ConvolGRU-MHA fusion model has a curve that closely fits the actual value curve, exhibiting better stability. As evident from Fig. 24, the overall error fluctuation of the proposed prediction model is the smallest, and the maximum error is 0.4%, demonstrating that the proposed ConvolGRU-MHA prediction model has better SOC prediction performance.

To further compare and analyze the predictive performance of the four algorithms, the Mean Absolute Error (MAE) and Root Mean Square Error (RMSE) were employed for comparison. Table 5 shows the RMSE and MAE of different prediction models. Based on Table 5, the proposed method applied in ConvolGRU-MHA model exhibits better predictive indicators than that of other models, such as GRU-MHA, ConvolGRU, and ConvolGRU-Attention. ConvolGRU-MHA prediction model has a smaller MAE and RMSE than the other three models, indicating that the proposed ConvolGRU-MHA prediction model has better SOC prediction performance.

**Table 5:** RMSE and MAE predicted by SOC of different models.

Model	RMSE	MAE
GRU-MHA	0.2466	0.1969
ConvolGRU	0.2438	0.1941
ConvolGRU-Attention	0.0218	0.0154
ConvolGRU-MHA	0.0067	0.0053

### Comparisons with other methods

Table 6 compares the proposed estimation performance to other model-based algorithms. These comparisons were conducted under similar or the same condition as this study.

**Table 6:** RMSE and MAE predicted by SOC of different models.

Ref	Method	RMSE	MAE (%)
Zhang et al^22^	LSTM-AT-Kalman	1.65%	1.49
Bian et al^23^	BLSTM-ED	/	1.07
Hannan et al^[﻿24^	GRU	0.96%	0.67
Ours	ConvolGRU-MHA	0.67%	0.53

Zhang considered using Kalman filter to modify the estimated result curve and improve the anti-interference performance of the network model. Bian Used BLstm to consider historical and future information, the bidirectional model constructed can capture the time information of LIBS from past and future directions, thus improving the estimation accuracy; Hannan changed the number of GRU hidden layers to improve the model's prediction performance. However, the feature information of the data is not fully extracted. In this paper, the convolution layer, GRU and multi-head attention mechanism are combined to extract data features from the two dimensions of time and space, and the data features extracted from the convolutional layer of multi-head attention mechanism and GRU are used to calculate the attention weight of variables, which can better identify the input variables related to SOC prediction. It can effectively reduce the prediction error of the model.

## Conclusions

This study proposed a ConvolGRU-MHA method for predicting the SOC of lithium-ion batteries. Two different evaluation functions, namely, MAE and RMSE, were employed to verify the accuracy of this proposed method. The predictive performance was also compared with that of GRU-MHA, ConvolGRU, and ConvolGRU-Attention models using a public dataset. Results indicate that The results show that the proposed model has the lowest RMSE of 0.67% and MAE of 0.53% compared with other predictive models. Compared to the single-fusion predictive models ConvolGRU and GRU-MHA, RMSE is reduced about 0.24 and MAE is reduced about 0.19. Compared to the ConvolGRU-Attention prediction model, RMSE was reduced 0.0151 and MAE was reduced 0.0101.The ConvolGRU-MHA model takes advantage of the powerful feature extraction capabilities of convolutional layers and GRU networks' ability to preserve the temporal data, allowing for comprehensive exploration of data features. The proposed model utilizes a multi-head attention mechanism, extracts more important features, and achieves effective and accurate prediction.

## Data Availability

The datasets used and analyzed during the current study are available from the corresponding author on reasonable request.
